# Cervical Lymph Node Metastasis in Chromophobe Renal Cell Carcinoma: A Case Report and Review of the Literature

**DOI:** 10.1155/2013/814175

**Published:** 2013-09-25

**Authors:** Noureddine Bouadel, Fahd El Ayoubi, A. Anass Bennani-Baiti, Mohamed Anas Benbouzid, Leila Essakalli, Mohammed Kzadri, Ali El Ayoubi

**Affiliations:** Department of Otorhinolaryngology, Head and Neck Surgery, Hospital des Spécialités, CHU Ibn Sina, Rabat, Morocco

## Abstract

The metastasis of chromophobe renal cell carcinoma to head and neck region, described herein, has never been reported before to our knowledge. A 56-year-old woman with a history of nephrectomy, that revealed chromophobe renal cell carcinoma six years before, presented left cervical mass. Imaging showed with left cervical lymphadenopathies and thyroid nodule. Surgery with histopathological examination confirmed that it was a left central and lateral jugular lymph node metastasis of chromophobe renal cell carcinoma treated postoperatively by antiangiogenic therapy. The patient was successfully treated by surgery and antiangiogenic drugs with stabilization and no recurrence of the metastatic disease. The case and the literature reported here support that chromophobe renal cell carcinoma can metastasize to the head and neck region and should preferentially be treated with surgery and antiangiogenic therapy because of the associated morbidity and quality-of-life issues.

## 1. Introduction

Renal cell carcinoma represents 3% of all adult malignant tumors [1]. Chromophobe renal cell carcinoma is a unique and infrequent subtype that comprises approximately 3% to 5% of all hypernephromas [2]. Although renal cell carcinoma is the third most common infraclavicular neoplasm that metastasizes to the head and neck region. Liver and lung are the most common sites of metastasis for chromophobe renal cell carcinoma that has better prognosis and lower potential to metastasize than the other subtypes of the hypernephroma [3, 4]. This case report and review of the literature describes a cervical lymph node metastasis in chromophobe renal cell carcinoma that has never been reported before in a large database search.

## 2. Patient and Methods

**Figure 1:**
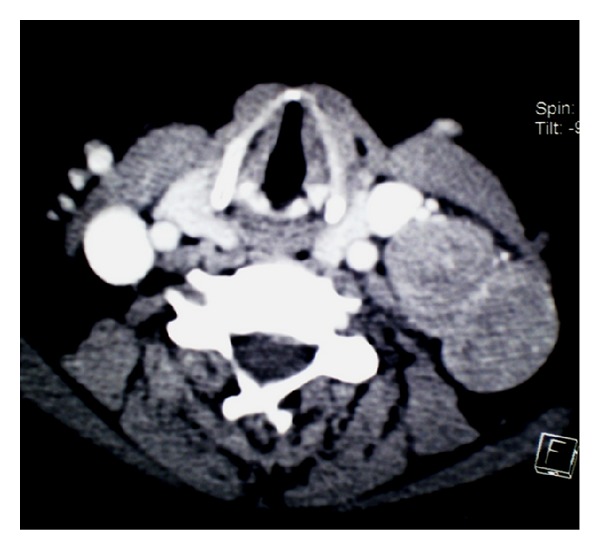
Contrast enhanced neck CT scan axial cup: left lateral jugular lymphadenopathy with heterogenic tissue density.

**Figure 2:**
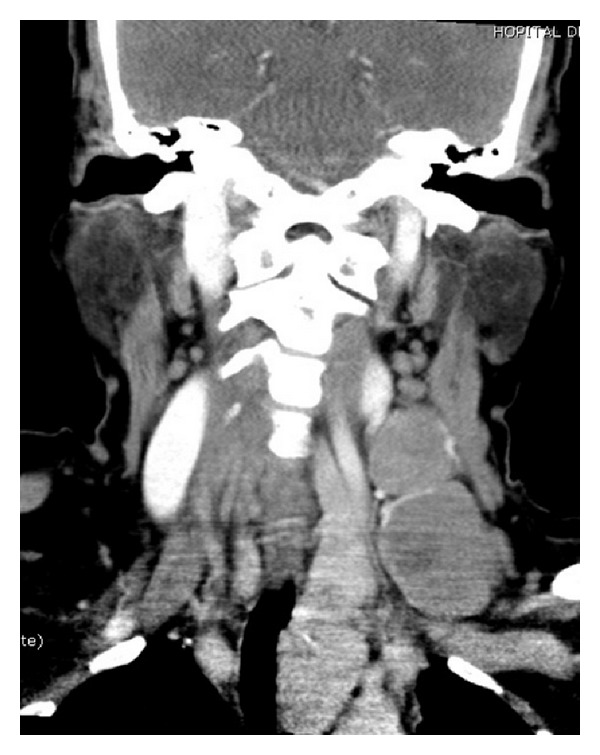
Contrast enhanced neck CT scan coronal cup: heterogenic left lateral tracheal tissue mass, probably a left diving thyroid nodule with 2 voluminous left lymphadenopathies with heterogenic density and no calcifications.

A 56-year-old female was admitted, in November 2011, to our department with a complaint of masse in her left neck region gradually evolved since 7 months with palpitations. The patient is asthmatic treated by Salbutamol, is hypertense with no treatment followed since 2009, and is treated for pulmonary tuberculosis 33 years ago. The past medical history of the patient included a left nephrectomy without therapeutic supplement in 2006 for renal tumoral syndrome revealed by left chronic lumbar pains. The pathological analysis revealed chromophobe renal cell carcinoma, T2N0Mx stage II, Fuhrman nuclear grade 2, and no sarcomatoid or papillary or tumor necrosis components were identified. A physical examination of the patient showed a painless left cervical masse; its greatest diameter is 04 cm with hard consistency. The thyroid function tests were normal. The thyroid ultrasonographic examination showed multinodular goiter with hypoechoic left nodule whose lower limit was flooded at the level of the Superior thoracic aperture. The ultrasonographic examination of the neck detected two contiguous left juguloomohyoid lymphadenopathies having a metastatic appearance. The CT scan of the neck showed a diving left thyroid nodule with left lateral jugular lymph nodes probably metastatic (Figures 1 and 2). An FNA biopsy from neck lymph node had not been made.

A total thyroidectomy was performed (extempore histopathological examination showed vesicular adenoma) with left functional posterolateral neck dissection (II, III, IV, and V levels) with anterior (or central) compartment neck dissection (VI) which found at left VIb a voluminous lymphadenopathy adherent to the left thyroid lobe lending confusion to a thyroid nodule. The pathological examination of the dissected lymphadenopathies in left VIb found the appearance of metastatic chromophobe renal cell carcinoma confirmed by immunohistochemical study. The pathological slides of the primary tumor were reviewed, and it was found that both the primary tumor and the neck metastases had the same origin.

**Figure 3:**
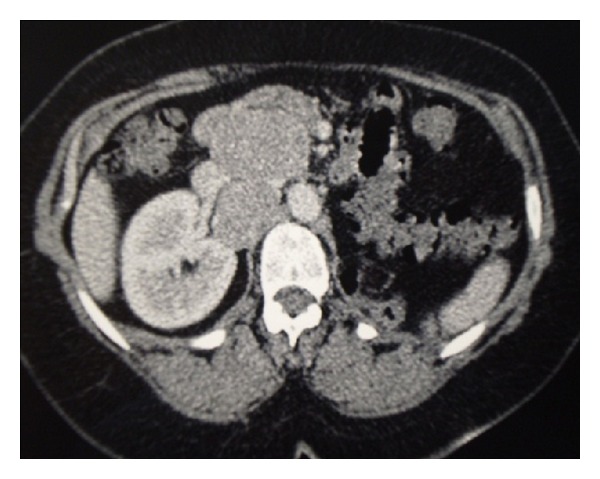
Contrast enhanced abdominal CT scan shows an empty renal lodge on the left side with voluminous retroperitoneal lymphadenopathies pre- and retroinferior cava rolling the inferior vena cava and the right renal vein.

An abdominal ultrasonographic examination showed a nephrectomy scar, on the left side and found retroperitoneal lymph nodes. The scintigraphic bone scan imaging was negative for metastatic lesions. The thoracoabdominopelvic scan also confirmed ultrasonographic findings and detected 3 voluminous retroperitoneal lymph nodes probably metastatic (Figure 3). 

Based on the above features, a decision was made consisting for no surgical treatment on the retroperitoneal nodes. The patient was started on antiangiogenic therapy post-operatively (sunitinib). After the sixth month of the operation and the second month of the first cure of sunitinib, she had no evidence of the recurrence of the neck region and another thoracoabdomino-pelvic CT scan confirmed that the retroperitoneal lymph nodes kept the same number and measures.

## 3. Discussion

Renal cell carcinoma (RCC) is rather a rare neoplasm and accounts for 3% of all human malignancies and usually occurs in men (male-female ratio: 1.5/1) between the ages of 30 and 60 years [1]. Approximately 200,000 new cases of RCC are diagnosed annually worldwide, while the number of deaths caused by RCC approaches 100,000. Cure can be obtained in 70–90% of patients in the TNM stage I, in 55–70% of patients in stage II, in 20–30% of patients in stage III, and in less than 10% in stage IV [5].

The 2004 World Health Organization (WHO) classification of RCC recognized several subtypes of RCC. Most common subtypes are: clear-cell RCC (70%), papillary RCC (10–15%), chromophobe RCC (4–6%), collecting duct carcinoma (about 1%), and unclassified RCC (4-5%) [5].

Distant metastasis of hypernephroma commonly occurs. The more frequent sites are the lungs (76%), the bones (42%), and the liver (41%). When the incidence of metastatic renal carcinoma to the head and neck region was reviewed, it was found that this type of tumor had been responsible for 14.3% of the metastases [3]. For eight percent of the patients, the presenting clinical manifestation of the tumor is disease in the head and neck. Only 1% of the patients with hypernephroma have no other obvious metastasis except that in the head and neck [6]. 

The clinical behavior of RCC is often unpredictable in its rate of growth, in the timing of metastasis, and in the variability of patterns of metastatic spread. Some hypernephromas have been regressed spontaneously, whereas others demonstrate metastasis many years after a supposedly curative nephrectomy (as the case of our patient). These metastases are usually vascular and may either clinically precede the diagnosis of the renal primary tumor or occur as long as 15 to 20 years after an apparently successful surgical excision of the primary tumor. 

The thyroid gland is the most common site of metastasis for this tumor in the head and neck region. The metastases can also occur in the cervical lymphatics, the mandible, the sinonasal tract, and the skin of the face and scalp. It has been postulated that some hypernephromas have the ability to bypass the pulmonary capillary filtration mechanism and metastasize directly to the head and neck region. One of the possible explanations for this phenomenon is tumor embolization by way of Batson's plexus of extensive anastomoses between the avalvular vertebral and epidural venous systems. Once the tumor emboli reach the head and neck region, they can anastomose with the great veins of the head and spread to the nose and sinuses, cutaneous sites, and thyroid gland [6]. 

It has also been postulated that the metastasis of renal cell carcinoma could also spread to the head and neck region through a normal hematogenous flow through the lungs, leaving microscopic seeding of the lung parenchyma, which would not be visible on a routine chest radiograph. Another theory postulates a lymphatic spread of metastatic embolus flowing to the regional lymphatics into the thoracic duct and arriving in the head and neck region by means of a retrograde flow through the intercostal, mediastinal or supraclavicular lymph vessels to the subglottis and above [6].

 The first cases of chromophobe renal cell carcinoma (CRCC) in humans were described in 1985 by Thoenes et al. [7]. In 1986, CRCC was included in the Mainz classification of renal cell tumors, which was based on histological and cytological criteria. The Mainz classification was later revised in the Heidelberg classification [8], which represents the current standard for subtyping renal cell tumors. Its frequency was evaluated as ranging from 3.6% to 10.4% of all kidney neoplasms [9, 10]. 

CRCC is diagnosed mainly in the sixth decade of life. An incidence of CRCC is similar in both men and woman. 86% of CRCCs are diagnosed in stage I or II. Renal vein invasion is seen in about 5% of cases. Incidence of metastatic disease in chromophobe renal cell carcinoma is 6-7%. In summary of 28 cases based on 7 reports, most common metastatic sites were liver (39%) and lung (36%). Metastasis to head and neck region of CRCC is exceptional [4]. 

Our patient is the only case published, having anterior and lateral cervical lymphatic node metastasis of CRCC (with no histopathological criteria of aggressive variant), essentially unilateral anterior (central) compartment and lateral jugular lymphatic levels. To our knowledge and based on a large bibliographic database search (PubMed, Hinari, Cochrane, Medline, Science Direct, Scopus, Google Scholar), metastasis in CRCC to head and neck region has never been reported before. 

 CRCC is a distinct type of renal cancer, is presumably derived from the intercalated cells of the collecting duct system, and exhibits a better prognosis than other types of RCC.

Macroscopically, CRCC is a solitary, circumscribed, and not capsulated mass with a homogeneous light brown cut surface. The median tumor size of CRCC is 6.0 cm, and it is larger than that of other subtypes. Microscopically, it contains large, polygonal cells with prominent cell membrane. Cytoplasm is pale and resistant to staining with hematoxylin and eosin. CRCC cells have irregular nuclei with perinuclear clear halo. The tumor blood vessels have thick walls and are eccentrically hyalinized [11].

**Figure 4:**
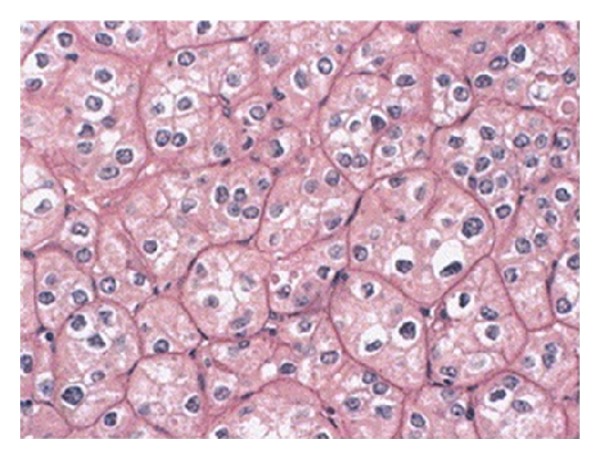
Chromophobe renal cell carcinoma on hematoxylin and eosin stain [12].

CRCC is a heterogeneous group including classic type, eosinophilic type, and mixed type (Figure 4). Eosinophilic variant (containing greater than 80% eosinophilic cells) has areas similar to renal oncocytomas (nested, alveolar, or shee-tlike architecture with eosinophilic granularity, perinuclear clearing, and peripheral accentuation of cytoplasm) and it is often bilateral (11%) and multifocal (22%). Classic type of CRCC (containing greater than 80% pale cells) is associated with necrosis or sarcomatoid change. It has alveolar or shee-tlike architecture and cytoplasm with flocculent “soap-bubble” appearance. CRCCs with mixed histology have variable architecture (containing admixture of pale and eosinophilic cells) [4].

Microscopic tumor necrosis and sarcomatoid change are known to be aggressive with a high potential for distant metastases. One of the diagnostic criteria of CRCC is Hale colloidal iron, another is intracytoplasmatic microvesicles between 250 and 400 nm in diameter. Otherwise, the main diagnostic criteria of CRCC are morphology coupled with characteristic immunophenotype (diffuse CK7 and KIT positivity) [4]. An inherited disorder, the Birt-Hogg-Dubé syndrome, has recently been described. This autosomal dominant condition is characterized by a familial tendency to develop multiple cutaneous fibrofolliculomas and trichodiscomas of the hair follicle. Additional studies have reported a predisposition for these families to develop multifocal or bilateral renal cancer, particularly CRCC [10].

 Prognosis in CRCC is better than that in other types of RCC. In the nomogram of Kattan et al. which predicts recurrence-free survival for patients with N0M0 disease, CRCC was considered to be less aggressive than papillary and clear-cell RCC. Cheville et al. and Beck et al. showed that the 5-year cancer-free survival rate was 86.7% and the disease-free survival rate was 80.1%. The median time from nephrectomy to metastasis detection and from metastasis detection to death was twice as long for CRCC than for other subtypes of RCC (papillary, clear cell RCC) [10]. 

Thus, the possible association with clear cell carcinoma, the existence of aggressive variants, and the difficulties in discriminating among these could justify an ablative radical treatment [10]. The role of surgery in metastatic renal cell carcinoma, especially the cytoreductive nephrectomy, has been source of controversy and usually is for diagnosis and debulking of disease. The metastasectomy is recommended when the localisation of the metastasis is unique and the surgery is technically possible [13]. Surgeons should be aware of the vascularity of these tumors when performing procedures [6]. However, the vascular nature of these metastases is not always clinically evident, as in our case. Eggener et al. showed that metastasectomy was an independent and significant factor to increase overall survival rates [14]. Pritchyk et al. consider head and neck metastases should be viewed differently because the lesion can lead to airway compromise, severe bleeding, and severe disfigurement [12]. Based on the presented case, we agree with them that depending on the site of presentation, local resection (functional neck levels dissection for cervical lymph node metastases, rarely radical dissection) may improve the quality of life and can provide a chance for cure in the head and neck. Furthermore, no standardization existed regarding the performance of the retroperitoneal lymph node dissection [8].

Renal cell carcinoma is traditionally described as a radioresistant tumor and responds relatively poorly to standard chemotherapeutic agents [15]. Metastatic CRCC seems to be a disease with a unique metastatic pattern and without response to immunotherapy. Despite immunotherapy, new therapeutic agents are now approved for the treatment of metastatic RCC including tyrosine kinase inhibitors such as sorafenib and sunitinib and the mTOR inhibitor temsirolimus. Klatte et al. recently presented data on safety and efficacy of sorafenib in patients with non-clear-cell RCC [8]. Stec et al. confirmed that sunitinib and sorafenib are active agents in metastatic CRCC: 75% of patients had stable disease (SD) more than 3 months and 25% had partial response (PR) [15].

Now we have three potentially active and targeted agents against KIT (CD 117): imatinib [KIT tyrosine kinase inhibitor (TKI)], dasatinib [second-line multikinase (besides BCR/ABL kinase) inhibitor], and nilotinib (the result of modifications to the imatinib molecule) [15].

## 4. Conclusions

The experience described herein confirms that bizarre sites of metastases from chromophobe renal cell carcinoma should be kept in mind by clinicians and surgeons. Also, chromophobe renal cell carcinoma, with or without histopathological aggressive criteria, can metastasize to head and neck region. Moreover, renal cell carcinoma should be considered in the differential diagnosis of any growing lesion in the head and neck and should preferentially be treated by surgery and antiangiogenic therapy.
